# The complete mitochondrial genome of *Buccinum bayani* (Gastropoda: Buccininae) in Korea

**DOI:** 10.1080/23802359.2022.2079098

**Published:** 2022-06-01

**Authors:** Kang-Rae Kim, Ho-Seop Han, Yong Hwi Kim, Jong Yeon Park, In-Chul Bang

**Affiliations:** Department of Life Science & Biotechnology, Soonchunhyang University, Asan, Republic of Korea

**Keywords:** *Buccinum bayani*, mitogenome, Buccininae, Gastropoda

## Abstract

This study is the first to report the complete 15,262 base pair mitochondrial genome of *Buccinum bayani*, consisting of 13 protein-coding genes, two ribosomal RNA genes, 22 transfer RNA genes, and a control region (D-loop). The overall base composition of the complete genome is A (29.87%), T (39.11%), G (15.86%), and C (15.16%), with a high AT content of 68.98%. Phylogenetic analysis showed that *B. bayani* was most closely related to *B. undatum*. The mitogenome of *B. bayani* will provide helpful information to further our phylogenetic and evolutionary understanding of the family Buccininae.

*Buccinum bayani* Jousseaume, 1883 is a gastropod that is harvested in Korea and Japan. It is found at depths of 100–400 m on the east coast of South Korea and is collected for commercial purposes. As an aquatic resource, it is a high-class gastropod and is very popular in Korea. However, there have been instances where *Buccinum tsubai*, which is similar to *B*. *bayani*, has been illegally sold as *B*. *bayani* to increase profits. Therefore, this study provides details of the mitogenome of *B*. *bayani* and initial data to develop species-specific markers. Our study will aid taxonomic understanding of the family Buccininae.

The *B*. *bayani* specimens in this study were caught in the East Sea (37°43′ N, 129°23′ E) of South Korea and purchased from a Korean market. A specimen was studied after morphological identification. *B*. *bayani* is an invertebrate and in accordance with the regulations of the Soonchunhyang University Institutional Animal Care and Use Committee (IACUC), invertebrates (gastropods) are exempt from research ethics approval and use for research. Some of the cut muscle tissue inside the shell was stored in 99% ethanol, and a voucher number was assigned in the specimen room of Soonchunhyang University, Asan, Chungcheongnam-do, Republic of Korea (Voucher Storage: Soonchunhyang University; Voucher number: SUC-26729; The person in charge of the collection: KR Kim; email: kimkangrae9586@gmail.com). Before genomic DNA extraction, mucus attached to the muscle tissue was thoroughly washed with triple-distilled water, and genomic DNA was extracted using a Genomic DNA Prep Kit (Biofact, Yuseong-Gu, Daejeon, Korea). The extracted genomic DNA was stored at −80 °C.

We used an MGI Easy DNA Library Prep Kit (MGI Technology, Shenzhen, China) and the extracted genomic DNA to produce a DNA library. Sequencing was performed with 150-base-pair (bp) paired-ends using the MGISEQ-2000 platform (MGI). *B*. *bayani* raw data were assembled using Geneious 11.0.3 (https://www.geneious.com). The fully assembled mitogenome sequence of *B*. *bayani* was annotated using the MITOS web server (Bernt et al. 2013) and deposited in the National Center for Biotechnology Information GenBank under accession number MW646292. The complete mitochondrial genome of *B*. *bayani* is 15,262 bp, containing 13 protein-coding genes (PCGs), two ribosomal RNA (rRNA) genes, 22 transfer RNA genes, and a control region (D-loop). Eleven PCGs, excluding the *CO3* and *ND4* genes, started with ATG. The stop codons of all PCGs terminated with TAA and TAG.

The overall base composition of the *B*. *bayani* genome is A (29.87%), T (39.11%), G (15.86%), and C (15.16%), with a high AT content of 68.98%. *B*. *bayani* rRNA comprised large and small rRNAs consisting of 1354 and 880 bp, respectively.

We used PhyML 3.0 to construct maximum-likelihood phylogenetic trees, and the GTR + I + R model proposed in jModelTest 2.1.10 (Guindon and Gascuel 2003; Guindon et al. 2010; Darriba et al. 2012) was applied based on 13 PCG sequences from 15 species.

Phylogenetic analysis showed that *B*. *bayani* was most closely related to *B*. *undatum* and grouped in the genus *Buccinum*. The mitogenome of *B*. *bayani* will provide helpful information to further our phylogenetic understanding of the genus *Buccinum* (Figure 1).

## Author contributions

The first author (Kim Kang-Rae) analyzed the data and wrote the final thesis. The second author (Han Ho-Seop) collected the data and wrote the thesis. The third author (Yong Hee Kim) designed the study. The fourth author (Jong Yeon Park) prepared the draft and collected the data. The corresponding author (Bang In-Cheol) interpreted and approved the data while checking the content of final version of the paper.

## Data Availability

The genome sequence data that support the findings of this study are openly available in GenBank of NCBI at (https://www.ncbi.nlm.nih.gov/) under the accession no. MW646292. The associated BioProject, SRA and Bio-Sample numbers are PRJNA755793, SRX11805387 and SAMN20841306, respectively. A phylogenetic tree was constructed using the maximum likelihood method based on 13 PCGs from 15 species, including *Buccinum bayani*. The accession number is listed after the scientific name.
